# Morroniside ameliorates sevoflurane anesthesia-induced cognitive dysfunction in aged mice through modulating the TLR4/NF-κB pathway

**DOI:** 10.17305/bb.2024.11433

**Published:** 2024-12-06

**Authors:** Jianxing Chen, Bo Peng, Wenqian Lin, Yinjun Mao, Yongsheng Wang

**Affiliations:** 1 Department of Anesthesiology, the First Affiliated Hospital, Fujian Medical University, Fuzhou, China; 2 Department of Anesthesiology, National Regional Medical Center, Binhai Campus of the First Affiliated Hospital, Fujian Medical University, Fuzhou, China; 3 Anesthesiology Research Institute, the First Affiliated Hospital, Fujian Medical University, Fuzhou, China; 4 Department of Anesthesiology, Chongqing Traditional Chinese Medicine Hospital, Chongqing, China; 5 Department of Pharmacy, National Regional Medical Center, Binhai Campus of the First Affiliated Hospital, Fujian Medical University, Fuzhou, China; 6 Department of Pharmacy, Zhongshan Hospital (Xiamen), Fudan University, Xiamen, China; 7 Xiamen Clinical Research Center for Cancer Therapy, Zhongshan Hospital (Xiamen), Fudan University, Xiamen, China

**Keywords:** Morroniside, Mor, postoperative cognitive dysfunction, POCD, sevoflurane, Sev, neuroinflammation, TLR4/NF-κB pathway

## Abstract

Morroniside (Mor) is a bioactive compound found in *Cornus officinalis* with anti-inflammatory, neuroprotective, and antioxidant properties. The prolonged use of sevoflurane (Sev) has been associated with the development postoperative cognitive dysfunction (POCD). This study aimed to elucidate the mechanism of action of Mor in improving cognitive function. A model of cognitive dysfunction induced by Sev was established in aged mice and tested for behavioral analysis using a water maze experiment. Histopathological changes and neuronal apoptosis in mouse hippocampus were observed by hematoxylin and eosin (HE) staining, Nissl staining, and TUNEL staining. ELISA and qRT-PCR were used to determine the levels of inflammatory factors. The phenotypic transformation of microglia in the hippocampal tissue was assessed using immunofluorescence, flow cytometry, and qRT-PCR. The interaction between Mor and Toll-like Receptor 4 (TLR4) was analyzed using molecular docking. Western blotting was used to identify the levels of apoptosis-related proteins, synapse-related proteins, and TLR4/NF-κB pathway proteins. Inhalation of Sev caused a notable reduction in learning and spatial memory in old mice, which was ameliorated by Mor in a dose-dependent manner. Mor inhibited neuroinflammation, modulated the polarization state of hippocampal microglia, promoted their polarization to the M2 type, and alleviated sev-induced hippocampal tissue damage and neuronal apoptosis. Notably, Mor binds well to TLR4 and reduce TLR4-positive expression. Mor blocked Sev-induced TLR4/NF-κB pathway activation in the hippocampal tissues, and the TLR4 agonist CRX-527 attenuated the effect of Mor. In conclusion, Mor blocks the TLR4/NF-κB pathway, reducing hippocampal tissue damage and neuroinflammation caused by Sev, which in turn improves cognitive impairment in aged mice.

## Introduction

Postoperative cognitive dysfunction (POCD) is associated with cognitive impairments related to learning, memory, attention, and orientation and is often accompanied by reduced performance in social activities [1, 2]. Statistically, the occurrence of POCD ranges from approximately 10%–54%, and the incidence rate of non-cardiac surgery in patients aged over 60 years is 25.8% a week after surgery and 10% three months after surgery [3, 4]. POCD has a serious impact on patients’ postoperative quality of life as well as heavy burdens and pressures on society, families, and healthcare. However, the causes and mechanisms of POCD remain unclear. Numerous researches have indicated that getting older and exposure to anesthesia are significant contributors to the onset of POCD [5, 6]. Therefore, exploring the connection between anesthesia and cognitive dysfunction is clinically important for the prevention and treatment of POCD.

The connection between anesthetic medications and POCD has been a focal point of interest in recent times, and the effects of anesthetic drugs on memory and consciousness in the human brain, such as type, dose, and exposure time, have become a hot research topic [7–9]. It has been shown that prolonged and repeated exposure of mice to volatile anesthetics may result in neuropathological alterations in the brain and persistent cognitive impairment [10]. Sevoflurane (Sev) is a widely utilized inhalation volatile anesthetic drug, known for its characteristics of rapid induction, fast awakening, and smooth anesthesia. It is widely used in anesthesia for elderly patients [11, 12]. However, similar to other volatile anesthetics, Sev is believed to exert diverse effects on tissues and organs, such as the central nervous, cardiovascular, and respiratory systems [13–15]. In addition, rats exposed to Sev exhibited alterations in memory and spatial learning abilities, along with a possible impairment in long-term memory. The way it works might be connected to damaging hippocampal neurons, promoting neuroinflammation, and triggering neuronal apoptosis [16, 17]. Therefore, finding novel and efficient drugs to improve cognitive dysfunction caused by Sev and exploring their possible mechanisms of action are important for the prevention of POCD.

Morroniside (Mor) is a cyclic enol ether terpene glycoside compound mainly found in the traditional Chinese medicine Cornus officinalis, with a variety of pharmacological effects [18]. Previous research has demonstrated that Mor exhibits highly robust antioxidant and anti-inflammatory properties, such as the ability to reduce interleukin-1β (IL-1β), tumor necrosis factor-α (TNF-α), and IL-6 levels and elevate glutathione and superoxide dismutase levels [19]. Notably, Mor ameliorated motor function and glial cell damage in Parkinson’s disease mice and inhibited nigral ferroptosis [20]. In addition, Mor promoted the transformation of microglia into the M2 phenotype in a murine model of middle cerebral artery occlusion, leading to elevated levels of IL-10 and a decrease in the size of the cerebral infarction [21]. It is thus evident that Mor protects the nervous system from injury, but so far, no report has been made on the impact of Mor treatment on POCD induced by Sev.

Toll-like receptor 4 (TLR4) is found on the surface of numerous cell types and is responsible for recognizing danger signals and activating immune responses, which produce large amounts of proinflammatory cytokines that disrupt immune homeostasis and accelerate the progression of inflammatory diseases [22]. NF-κB is a crucial transcription factor that is activated after cells are stimulated by various substances, such as inflammatory mediators, viral infections, and oxidative stress, and is responsible for managing the innate and adaptive immune responses [23]. Numerous studies have demonstrated the significant involvement of the TLR4/NF-κB pathway in both cognitive decline and neuroinflammation [24, 25]. Therefore, in this study, a cognitive impairment model in aged mice induced by Sev was established to investigate the effect of Mor on cognitive impairment in mice. On this basis, it was further explored whether Mor alleviated cognitive impairment caused by Sev through hindering the TLR4/NF-κB pathway. The purpose of this study was to uncover how Mor improves cognitive impairment in mice and offers a new reference for treating POCD in clinical settings.

## Materials and methods

### Model construction and processing

Male SPF-grade C57BL/6J mice (20 months old) were acquired from the Vital River Laboratory Animal Technology Co., Ltd. (Beijing, China) and kept in a clean-grade animal house. room temperature was 23 ^∘^C–28 ^∘^C, with humidity levels between 45% and 55%, under a 12-h light–dark cycle, and feeding and drinking were performed autonomously. Animal experiments followed the 3R principle, changing bedding daily and disinfecting facilities, such as food containers, cages, water bottles, and drinking tubes regularly. After a week of adjustment feeding, mice were randomly divided into control (*n* ═ 8), Sev (Sev, *n* ═ 8), Sev-Mor (S-Mor, *n* ═ 16), and Sev-Mor-TLR4 agonist (S-Mor-CRX-527, *n* ═ 4) groups. An anesthesia gas monitor (Datex-Ohmeda Inc., Madison, WI, USA) was used to monitor the levels of Sev, carbon dioxide, and oxygen levels. A small amount of soda lime was spread on the bottom of the induction box to prevent carbon dioxide accumulation. Breathable isolation pads were laid flat on top of the soda-lime to prevent mice from inhaling soda-lime dust to burn the mice. A mouse model of cognitive dysfunction under Sev anesthesia was established according to Ma et al. [26]. Mice continuously inhaled 2% Sev for 5 h, with a total of 1.5 L/min airflow achieved using 70% O_2_ as the carrier gas. After the end of Sev anesthesia, they were returned to their cages after awakening in a dry and warm environment. Control group: normal inhalation of room air.

Control mice were intraperitoneally injected with saline (0.1 mL/100 g). After anesthetization, mice in the Sev group were intraperitoneally injected with saline. The mice in the S-Mor group were anesthetized before receiving Mor (30, 60, and 100 mg/kg body weight, HY-N0532, MedChemExpress, Monmouth Junction, NJ, USA) at a frequency of every three days over a period of four weeks. Mice in the S-Mor-CRX-527 group were anesthetized and injected intraperitoneally with Mor (100 mg/kg bw) every three days for four consecutive weeks, followed by intraperitoneal injection of CRX-527 (0.25 mg/kg bw, V42156, InvivoChem LLC, Shanghai, China) once a day for three days during the last three days of Mor treatment. The Ethics Committee for Animal Experiments of the First Affiliated Hospital, Fujian Medical University granted approval for the precise protocol and implementation of this study.

### Behavioral analysis tests

Mice were subjected to the Morris water maze test to assess their spatial memory capacity. The circular pool used in the water maze experiment measured 100 cm in diameter and 30 cm in depth and featured a platform that could be easily disassembled. The pool was divided into four equal parts labeled quadrants I, II, III, and IV, with a small white circular platform (7-cm wide) positioned at the center of quadrant I. The pool was surrounded by a reference, which remained unchanged. In the test, a white nontoxic dye was poured into the water until the platform could not be observed with the naked eye. The light within the space remained consistent, while the water temperature was maintained within the range of 22 ^∘^C–25 ^∘^C. The mice entered the water from different quadrants, and the escape latency was the period taken by the mice to enter the water, locate the hidden platform, and ascend onto it. The recorded result of the experiment was 60 s if the mice were unable to find the hidden platform within the time limit. The researchers recorded the mean path length taken by the mice starting from their entry into the water until they discovered the hidden platform and employed a timer to measure the duration that the mice stayed in the designated quadrant. Four tests were performed daily for four consecutive days. The concealed platform was removed on the fifth day, the starting position was randomly selected, the mice were introduced into the water, and the number of times within one minute that the mice passed through the position where the hidden platform was originally located was recorded. Swim speed was computed using the formula: distance × number of passes through the hidden platform (n)/60 s.

### Hematoxylin and eosin (HE) staining

After the behavioral analysis tests were completed, the mice were sacrificed on day 3, brain tissue was removed, and hippocampal tissue was obtained by dissection on ice. An HE staining kit (G1120, Solarbio, Beijing, China) was used to assess the lesions in the hippocampal tissue of mice. After treatment with 4% paraformaldehyde (P0099, Beyotime, Shanghai, China) for 24 h, hippocampal tissues were dehydrated in graded ethanol (100%, 95%, 75%, and 50%), paraffin-embedded, sectioned (4 µm), and the slices were baked for 8 h at 65 ^∘^C. After rinsing with distilled water, the samples were deparaffinized with xylene (247642, Sigma-Aldrich, St. Louis, MO, USA). The tissue samples were subjected to hematoxylin staining for 8 min, treated with differentiation solution (C0161s, Beyotime) for 30 s, and subsequently stained with eosin for 1 min. The samples were then sequentially dehydrated in different concentrations of ethanol, made transparent in xylene, sealed with neutral gum, and finally viewed using an inverted microscope (DM IL LED, Leica, Heidelberg, Germany).

### TUNEL staining

Wax blocks of mouse hippocampal tissue were routinely sectioned, treated with xylene to remove paraffin, and dehydrated with gradient ethanol for 5 min each. DNase-free proteinase K (20 µg/mL, ST532, Beyotime) was slowly introduced and the process was conducted for half an hour, followed by three PBS washes. Next, TUNEL assay solution (C1086, Beyotime) was added dropwise to evenly cover the tissues and incubated for 1.5 h away from light. Subsequently, the cells were incubated with DAPI staining solution (D9542, Sigma-Aldrich) for 10 min in the dark. After sealing with an anti-fluorescence quenching solution (HY-K1042, MedChemExpress), an inverted fluorescence microscope was used for observation and photography.

### Nissl staining

Wax blocks of mouse hippocampal tissue were sectioned and deparaffinized before dehydration with an ethanol gradient. Nissl staining solution (C0117; Beyotime) was added dropwise and kept in at 37 ^∘^C incubator for 10 min. Distilled water was washed twice and then dehydrated with 95% ethanol for 2 min. After xylene transparency for 5 min and neutral gum sealing, the samples were observed under an inverted microscope.

### ELISA

Mouse hippocampal tissue blocks were cleaned, cut with tissue shears, and ground in precooled PBS. After adequate grinding and ultrasonic crushing, the resulting tissue homogenate was centrifuged and the liquid portion was collected. ELISA was used to quantify inflammatory factors in hippocampal tissues. Mouse TNF-α (ml002095), IL-6 (ml098430), and IL-1β (ml098416) kits were purchased from Enzyme Link Biotechnology (Shanghai, China). The supernatant was introduced into an ELISA plate and incubated for 2 h. After adding PBS for three washes with PBS, the corresponding antibody was added and incubated for 1 h. Horseradish peroxidase-labeled streptavidin was then introduced and incubated for 20 min away from light. Substrates A and B were then added and incubated for 30 min. The termination solution was introduced and mixed thoroughly, the OD_450_ value was assessed, and the concentration was calculated.

### qRT-PCR

Mouse hippocampal tissues were ground on ice and lysed using TRIzol reagent (R0016, Beyotime). The RNA level in each sample was quantified as 0.2 µg/µL in to volume of 20 µL. cDNA was obtained by adding AMV reverse transcriptase (2621; TAKARA) for reverse transcription. The target gene was amplified using TB Green FAST qPCR (CN830S, TAKARA) in a PCR reaction with cDNA as a template. GAPDH was used as an internal standard, and the relative levels of the target genes were determined using the 2^−ΔΔCt^ method.

The primer sequences used were as follows: TNF-α, F: 5′-GGAAGAGGTGAGTGCCTGG-3′ and R: 5′-GCCCTGAGGTGTCTGGTTTT-3′. IL-1β: F: 5′-GGGGCGTCCTTCATATGTGT-3′; R: 5′-GGCAGCTCCTGTCTTGTAGG-3 IL-6: F: 5′-GCTTCCCTCAGGATGCTTGT-3′; R: 5′-ATTAACTGGGGTGCCTGCTC-3′. CD206: F: 5′-ACCAACCCCCACCACTTTATTT-3′; R: 5′-AGAGCCCTTGGGTTGAGGAT-3′. CD86: F: 5′-CTTCCACATTGCCCCGGTAT-3′; R: 5′-TGGAAATGCCCACCTACCAG-3′. Arginase-1(Arg-1): F: 5′-ACCTGAAACCAAGTCCCAGC-3′; R: 5′-CGAGCAAGTCCGAAACAAGC-3′. Inducible nitric oxide synthase(iNOS): F: 5′-CAGCATGAGCCCCTTCATCA-3′; R: 5′-TGAAGTCTGTGTCCGAAGGC-3′. GAPDH: F: 5′-TCGGCAGGATGTAGGGCTAAAAGC-3′; R: 5′-GTAGCCCATGGGTTTTAGCCC-3′.

### Immunofluorescence

Hippocampal tissue wax blocks were sectioned and dewaxed for microwave antigen repair. Tissue sections were permeabilized with Tritonx-100 (0.3%, X100, Sigma-Aldrich) for 10 min. Subsequently, the tissues were evenly covered with drops of 5% bovine serum albumin (BSA; V900933, Sigma-Aldrich) and closed for 30 min. Placed at 4 ^∘^C for an overnight incubation with glial fibrillary acidic protein (GFAP) antibody (14-9892-82, 1:50, Invitrogen, Carlsbad, CA, USA), ionized calcium-binding adapter molecule 1 (Iba-1) antibody (ab178846, 1:100, Abcam, Cambridge, MA, USA), TLR4 antibody (53-9917-42, 1:200, Invitrogen), or NF-κB p65 antibody (51–0500, 1:500, Invitrogen). The following day, the sections were exposed to FITC-labeled goat anti-rabbit IgG (F-2765, 1:1500, Invitrogen) in darkness for 1 h. Sections were then exposed to the DAPI staining solution (C1005, Beyotime) and incubated for 10 min, and the development was observed by fluorescence microscopy within 1 h after blocking.

### Flow cytometry

Mouse unilateral hippocampal tissue was obtained, carefully cut clean with tissue scissors, gently stirred with tweezers on a 100-mesh copper mesh, rinsed with PBS, and filtered to obtain a cell suspension. The cell suspension was filtered using a 300-mesh nylon mesh to remove large clumps, followed by centrifugation, and the supernatant was discarded. DMEM (11965092, Gibco, Grand Island, NY, USA) was added and mixed well so that the final concentration of the single-cell suspension was approximately 2×10^5^/mL. FITC-labeled CD11b antibody (11-0112-41, Invitrogen), PE-labeled CD16 antibody (12-0167-42, Invitrogen), CD86 antibody (12-0862-82, Invitrogen), CD163 antibody (12-1639-42, Invitrogen), and CD206 antibody (12-2061-82, Invitrogen) were mixed well and incubated for 30 min in a dark environment. Flow cytometry (BD FACSCaliburTM, BD Biosciences, San Jose, CA, USA) was used for detection, and the experimental data were analyzed using FlowJo software (v10.8, BD Biosciences).

### Molecular docking

The 3D configuration of Mor was sourced from the PubChem database (https://pubchem.ncbi.nlm.nih.gov/), whereas the TLR4 protein structure was retrieved from the RCSB PDB database (https://www.rcsb.org/). AutoDock software was used to confirm the molecular docking of Mor with TLR4. The general agreement is that docking energy values lower than −4.25 kcal/mol imply some form of binding interaction, while values below −5.0 kcal/mol signify a good binding interaction, and values below −7.0 kcal/mol represent a strong binding interaction.

### Western blot

Mouse hippocampal tissues were thoroughly ground, RIPA lysis buffer (P0013B, Beyotime) was added to obtain proteins, and the BCA kit (P0012, Beyotime) was used to assess protein concentrations. Protein samples were electrophoresed on SDS-PAGE gels (10%, Invitrogen) and then transferred to a PVDF membrane (Invitrogen), followed by blocking with BSA for 3 h. After rinsing, the membranes, placed at 4 ^∘^C with an overnight antibodies: cleaved caspase-3 primary antibody (PA5-114687, 1:2000, Invitrogen), Bax primary antibody (MA5-14003, 1:1000, Invitrogen), Bcl-2 primary antibody (ab59348, 1:500, Abcam), presynaptic growth-associated protein 43 (GAP-43) primary antibody (33-5000, 1:1000, Invitrogen), synaptosomal-associated protein-25 (SNAP-25) primary antibody (PA1-740, 1:200, Invitrogen), neurofilament light chain (NfL) primary antibody (13-0400, 1:200, Invitrogen), synaptophysin (Syp) primary antibody (MA5-14532, 1:200, Invitrogen), TLR4 primary antibody (53-9917-42, 1:1000, Invitrogen), NF-κB p65 primary antibody (51-0500, 1:400, Invitrogen), or p-p65 primary antibody (MA5-15160, 1:1000, Invitrogen). The following day, after three rinses, the membranes were exposed to goat anti-rabbit secondary IgG (31460, 1:10,000, Invitrogen), developed, and exposed. The ECL chemiluminescent agent (34577, Invitrogen) was evenly dispersed on the membranes and observed using a gel imaging system (iBright CL1500, Invitrogen). Image J software (version 1.54h, Wavne Resband, National Institute of Mental Health, USA) was used to process the images and obtain the grayscale values of each protein band, and the relative protein level was quantified by comparison with GAPDH (MA5-15738, 1:1000, Invitrogen).

### Ethical statement

The experimental animal protocol was approved by the Experimental Animal Ethics Committee of the First Affiliated Hospital of Fujian Medical University.

### Statistical analysis

A minimum of three repetitions were performed in each experiment, and the results are reported as the mean ± standard deviation. For statistical analysis of the data and image plotting, SPSS software (version 26.0; IBM SPSS Statistics 26) and Prism software (GraphPad 9.0) were used. Student’s *t*-test was used to evaluate the differences between the two groups, and analysis of variance (ANOVA) was performed to compare multiple subgroups. *P* < 0.05 demonstrated that the differences were considered statistically significant at *P* < 0.05.

## Results

### Mor ameliorates learning and memory deficits caused by Sev in aged mice

**Figure 1.**
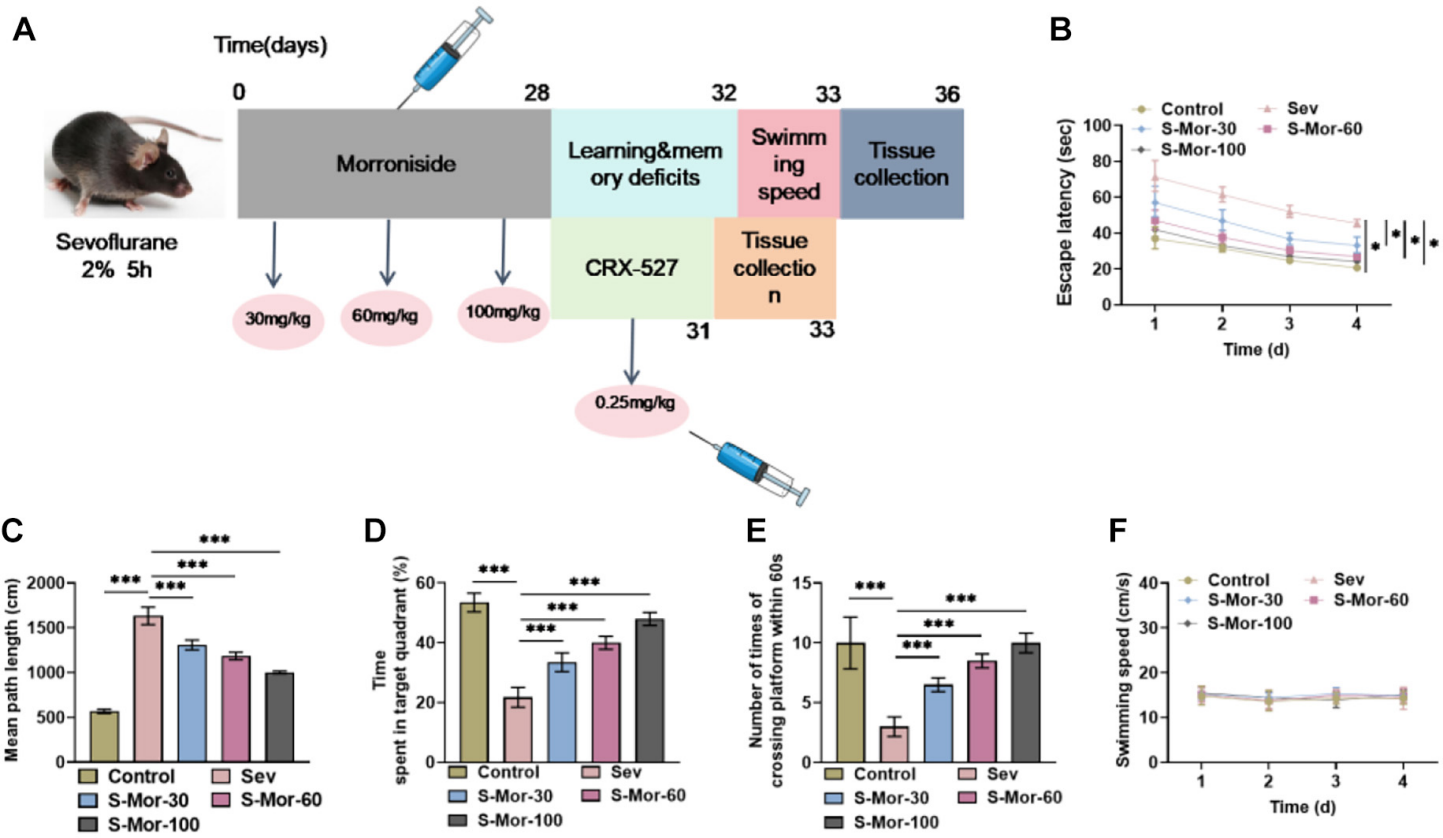
**Mor ameliorates Sev-induced learning and memory deficits in aged mice.** (A) Flowchart of mouse model construction and drug administration. The spatial memory capacity of mice was evaluated through the Morris water maze test, and the escape latency (B), mean path length (C), and time spent in the target quadrant (D) were recorded. The times of the mice traversed the platform in one minute was recorded (E), and their swimming speed was calculated (F). *n* ═ 4. (*P < 0.05, ***P < 0.001). Mor: Morroniside; Sev: Sevoflurane.

The cognitive impairment model in aged mice induced by Sev was constructed according to the procedure shown in Figure 1A by intraperitoneal injection of different concentrations of Mor every 3 d for 28 d, followed by behavioral analysis tests, and finally hippocampal tissues were collected. The findings of the water maze experiments indicated that continuous inhalation of 2% Sev for 5 h significantly prolonged the avoidance latency and mean path of aged mice, whereas the injection of Mor caused a marked shortening of the avoidance latency and mean path; the higher the concentration, the more pronounced the effect (Figure 1B and 1C). In addition, inhalation of Sev significantly shortened the time mice stayed in the spent quadrant and notably decreased the number of passes in one minute through the location where the covert platform was originally located, while the Mor treatment significantly lengthened the stay time and increased the number of passes through the platform location (Figure 1D and 1E). This suggests that Mor can significantly improve cognitive and spatial memory abilities in elderly mice. Notably, the swimming speed of the elderly mice was not affected by either Sev or Mor (Figure 1F).

### Mor inhibits histopathological damage in the hippocampus of aged mice

**Figure 2.**
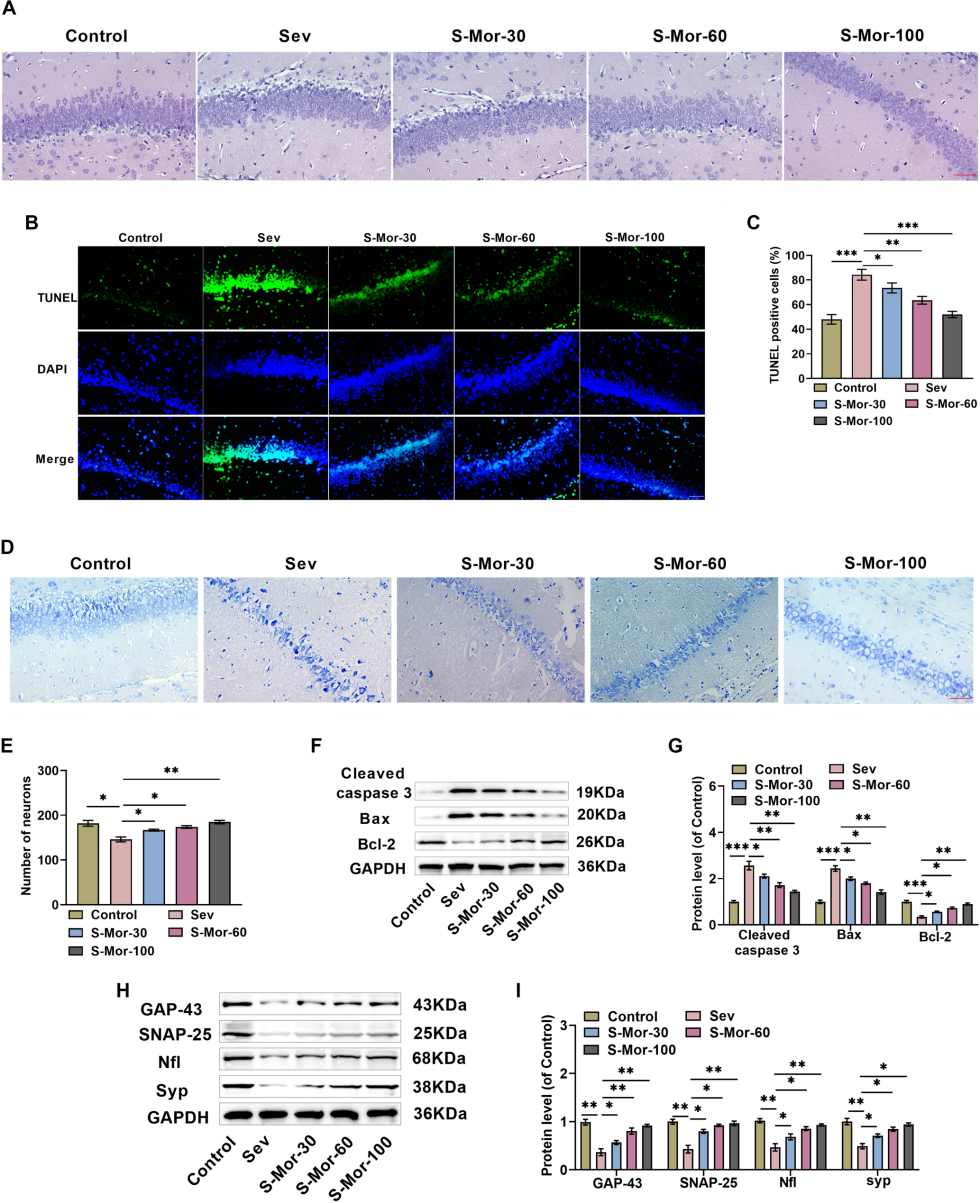
**Mor inhibits histopathological damage caused by Sev in the hippocampus.** (A) Three days following the conclusion of the behavioral analysis test, mice were executed, brain tissue was removed, and hippocampal tissue was obtained by dissecting on ice. Pathologic changes in mouse hippocampus were observed through HE staining; (B and C) Detection of hippocampal neuronal apoptosis by TUNEL staining; (D and E) The quantity of neurons in the mouse hippocampus was identified through Nissl staining; (F and G) Examining the levels of cleaved-caspase 3, Bax, and Bcl-2 levels in hippocampal tissue through western blot; (H and I) Western blot was employed to evaluate the levels of neural-related proteins Gap-43, Snap-25, Nfl, and Syp in hippocampal tissues. *n* ═ 4. (*P < 0.05, **P < 0.01, ***P < 0.001). Mor: Morroniside; Sev: Sevoflurane; HE: Hematoxylin and eosin.

Next, we examined how Sev and varying levels of Mor treatment affected histopathological damage in the hippocampus. HE staining revealed that the hippocampal cells of the control mice were regular in shape, relatively neatly arranged, with clear boundaries, consistent cytoplasmic staining, obvious nuclear staining, and no obvious structural abnormalities were observed. After inhalation of Sev, mouse neurons were atrophic and degenerated, with dispersed cell arrangement, obvious interstitial edema, and unclear intercellular boundaries, whereas Mor treatment significantly ameliorated sev-induced hippocampal cell damage in a concentration-dependent manner (Figure 2A). TUNEL staining analysis indicated a notable increase in the number of TUNEL-positive neuronal cells in aged mice after inhalation of Sev, and the injection of Mor decreased the number of TUNEL-positive cells, suggesting that Mor ameliorated Sev-induced neuronal apoptosis (Figure 2B and 2C). In addition, Nissl staining findings showed that Sev caused a significant decline in the quantity of neurons in elderly mice, which was dose dependently ameliorated by Mor (Figure 2D and 2E). In addition, western blot findings indicated a notable increase in apoptosis-associated protein cleaved-caspase 3 and Bax levels, along with an elevation in the anti-apoptotic protein Bcl-2 within the hippocampal tissues of old mice after Sev inhalation, whereas the injection of Mor alleviated the abnormal expression of these proteins (Figure 2F and 2G). Notably, Sev caused a notable decrease in the levels of synapse-related proteins Gap-43, Snap-25, Nfl, and Syp, whereas injection of Mor increased the levels of these proteins (Figure 2H and 2I). These findings suggest that Mor can alleviate sev-induced hippocampal tissue damage and neuronal apoptosis in aged mice and improve synaptic dysfunction.

### Mor inhibits Sev-induced neuroinflammation

**Figure 3.**
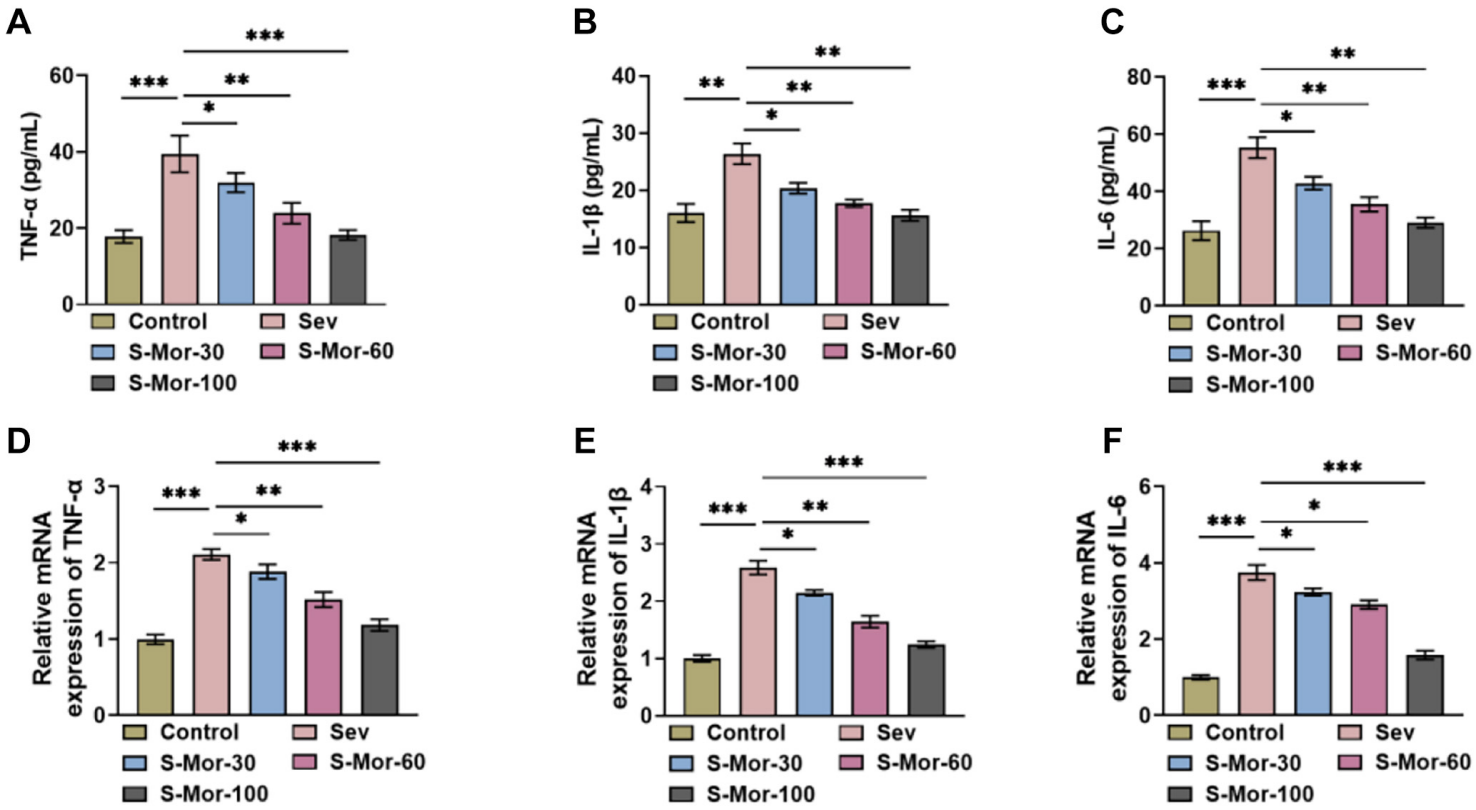
**Mor inhibits Sev-induced neuroinflammation.** (A–C) The levels of cytokines TNF-α, IL-1β and IL-6 were assessed by different ELISA kits and (D–F) qRT-PCR was utilized to quantify TNF-α, IL-1β and IL-6 expression in hippocampal tissues. *n* ═ 4. (*P < 0.05, **P < 0.01, ***P < 0.001). Mor: Morroniside; Sev: Sevoflurane.

ELISA findings indicated that inhalation of Sev caused a notably rise in TNF-α, IL-1β and IL-6 levels in the hippocampal tissues of aged mice, whereas Mor decreased the concentrations of inflammatory cytokines in a manner that depended on the dosage (Figure 3A–3C). In addition, the expression of these inflammatory factors was examined by qRT-PCR, and the levels of TNF-α, IL-1β, and IL-6 expression were markedly upregulated by inhalation of Sev, whereas Mor reduced excessive inflammatory cytokine expression (Figure 3D–3F). These findings indicated that Mor can effectively reduce Sev-triggered neuroinflammation.

### Mor promotes the shift of hippocampal microglia from M1 to M2 phenotype in cognitively impaired mice

**Figure 4.**
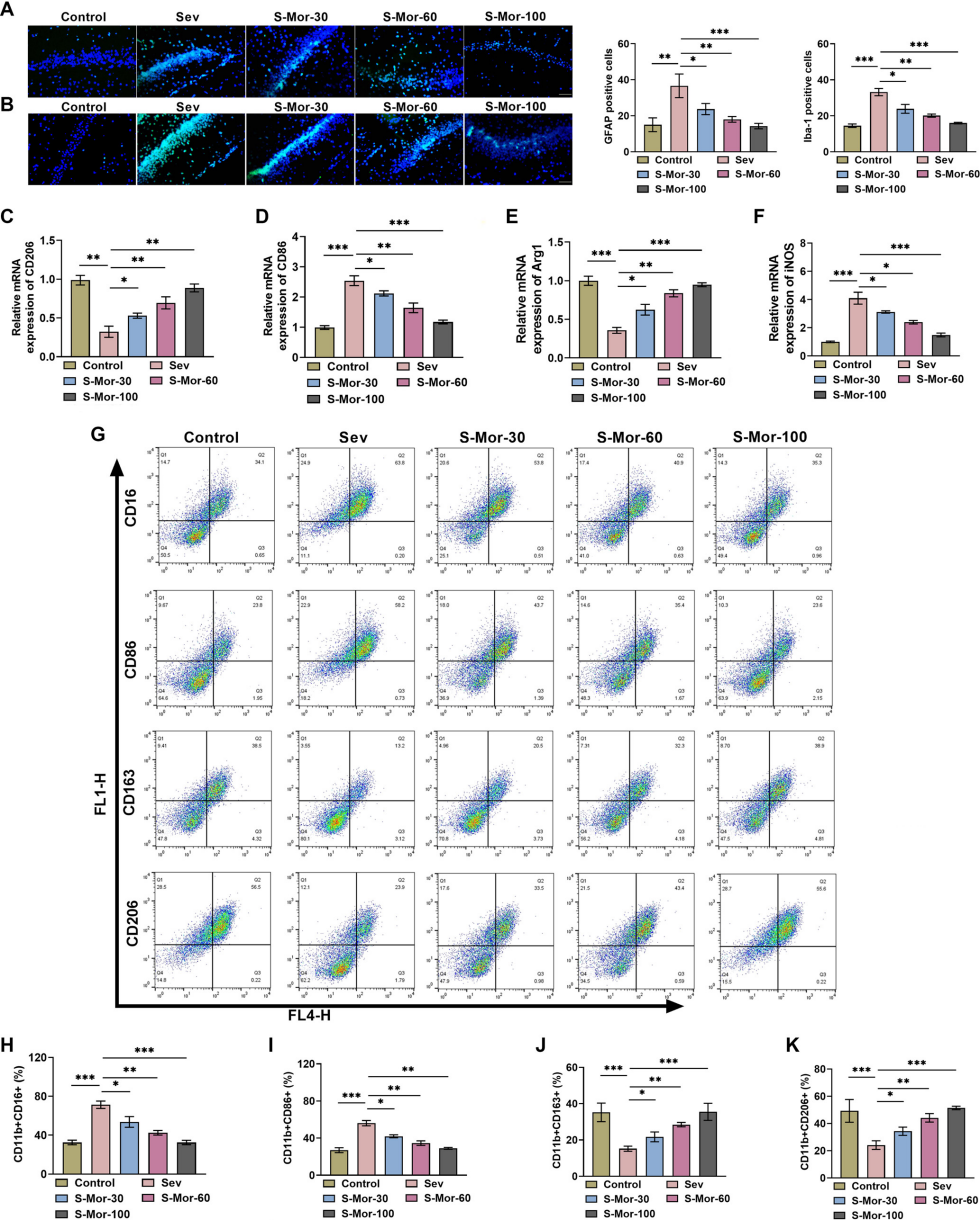
**Mor promotes the transition of hippocampal microglia from M1 to M2 type in cognitively impaired mice.** (A and B) Positive expression of GFAP and Iba-1 was assayed by immunofluorescence; (C–F) qRT-PCR detected CD206, CD86, Arg1, and iNOS expression levels in hippocampal tissues; (G) Flow cytometry determined the proportions of CD11b + CD16+ (H), CD11b + CD86+ (I), CD11b + CD163+ (J), and CD11b + CD206+ (K) cells in hippocampal tissue. *n* ═ 4. (*P < 0.05, **P < 0.01, ***P < 0.001). Mor: Morroniside.

To explore the specific mechanisms by which Mor ameliorates cognitive deficits in aged mice, we assessed the effects of Mor on phenotypic shifts in hippocampal microglia. GFAP and Iba-1 are common markers of astrocyte and microglia [27]. By immunofluorescence, it could be observed that inhalation of Sev caused a notable rise in the quantity of GFAP-positive cells and Iba-1-positive cells in the CA1 region of the mouse hippocampus, suggesting that Sev induces glial cell activation, whereas Mor injection markedly declined the number of cells positive for GFAP and Iba-1 (Figure 4A and 4B). The levels of M2 polarization markers (CD206 and Arg1) in the hippocampal tissues of aged mice were significantly reduced after inhalation of Sev, while M1 polarization markers (CD86 and iNOS) were significantly elevated, and the injection of Mor promoted the shift of hippocampal microglial cells to the M2 type (Figure 4C–4F). Not only that, flow cytometry results also showed that Sev caused a notable rise in the proportion of M1-type cells (CD11b + CD16+, CD11b + CD86+) and a marked decline in the proportion of M2-type cells (CD11b + CD163+, CD11b + CD206+), whereas Mor injection caused a notable reduction in the ratio of M1-type cells and a rise in the proportion of M2-type cells (Figure 4G–4K). The above results confirm that Mor promotes the shift of hippocampal microglia from the M1 to M2 type in Sev-induced cognitively impaired mice.

### Mor inhibits TLR4 activation in hippocampal tissue of cognitively impaired mice

**Figure 5.**
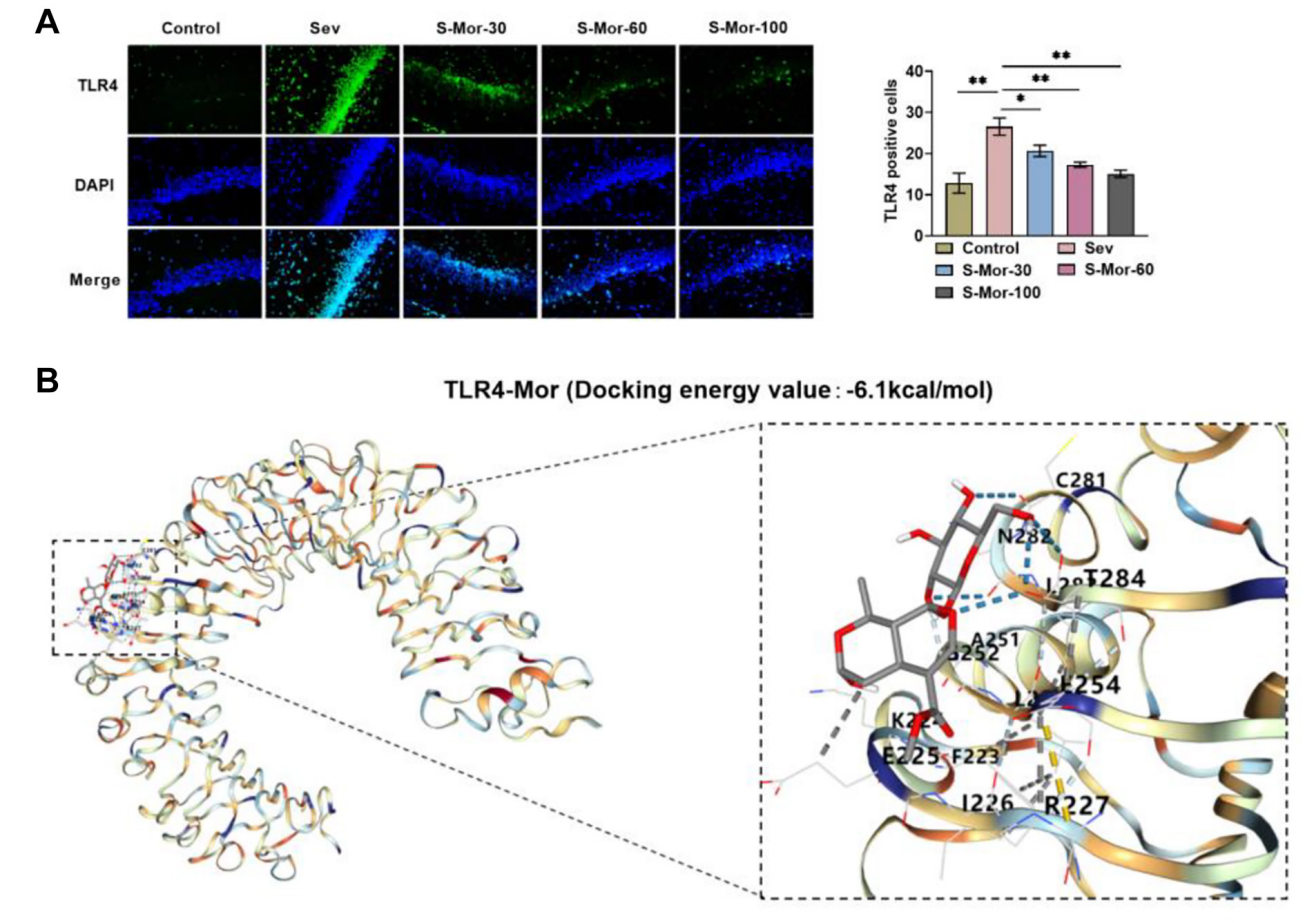
**Mor inhibits TLR4 activation in hippocampal tissue of cognitively impaired mice.** (A) Immunofluorescence staining detected positive TLR4 expression in the hippocampus and (B) The binding of TLR4 and Mor was verified by molecular docking. *n* ═ 4. (*P < 0.05, **P < 0.01). Mor: Morroniside; TLR4: Toll-like receptor 4.

TLR4, an important transmembrane receptor, is crucial for the immune response in the body [28]. To further explore the molecular mechanisms by which Mor ameliorates cognitive impairment, we assessed the effect of Mor on TLR4. The number of TLR4-positive cells in hippocampal tissues was notably elevated after inhalation of Sev, and injection of Mor dose-dependently decreased TLR4-positive expression (Figure 5A). Notably, by molecular docking, we found that Mor and TLR4 can bind as hydrogen bonds and the binding energy is −6.1 kcal /mol, which is well-bound (Figure 5B). This suggests that Mor inhibits its activation through targeted binding to TLR4, which in turn alleviates neuroinflammation and promotes microglial phenotypic transformation.

### Mor inhibits TLR4/NF-κB pathway activation in hippocampal tissues of Sev-treated aged mice

**Figure 6.**
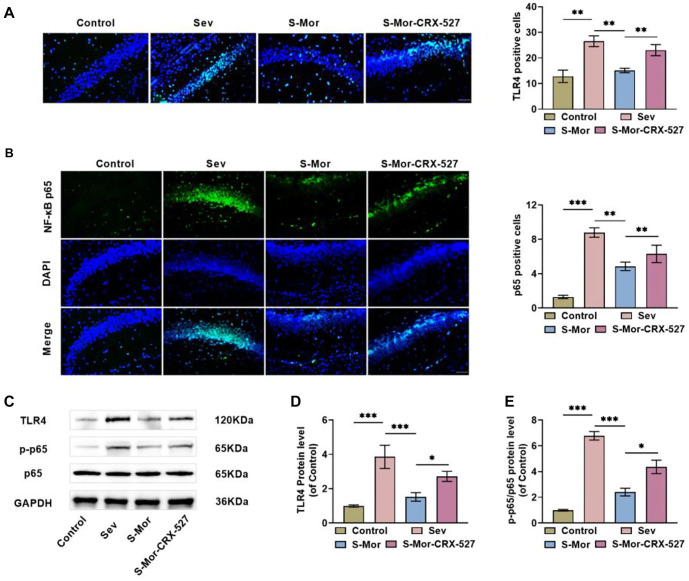
**Mor inhibits TLR4/NF-κB pathway activation in hippocampal tissue of Sev-treated aged mice.** (A and B) Positive expression of TLR4 and nuclear localization of NF-κB p65 in hippocampal tissue detected by immunofluorescence; (C–E) Examining the levels of TLR4/NF-κB pathway protein levels in hippocampal tissue through western blot. *n* ═ 4. (*P < 0.05, **P < 0.01, ***P < 0.001). Mor: Morroniside; Sev: Sevoflurane; TLR4: Toll-like receptor 4.

Finally, to assess whether Mor improves cognitive deficits in aged mice by suppressing TLR4 signaling, we intraperitoneally injected the TLR4 agonist CRX-527 into mice. Immunofluorescence findings revealed that the number of TLR4-positive cells was notably elevated after the injection of CRX-527, indicating that CRX-527 reversed the inhibitory effect of Mor (100 mg/kg body weight) on TLR4 and satisfied the requirements for subsequent experiments (Figure 6A). NF-κB acts as a pivotal downstream factor of TLR4 signaling, and inhalation of Sev caused a notable elevation in the quantity of NF-κB p65-positive cells in mouse hippocampal tissues, and injection of Mor decreased the quantity of NF-κB p65-positive cells, while CRX-527 attenuated the effect of Mor (Figure 6B). Not only that, after inhalation of Sev, TLR4 protein and NF-κB p65 phosphorylation levels were markedly elevated in mouse hippocampal tissues, and Mor treatment inhibited Sev-induced activation of the TLR4/NF-κB pathway, whereas the inhibitory impact of Mor was attenuated by CRX-527 (Figure 6C–6E). In conclusion, activation of the TLR4/NF-κB pathway attenuated the effects of Mor treatment, suggesting that Mor improves cognitive deficits by hindering the TLR4/NF-κB pathway.

## Discussion

Sev is a commonly employed inhalation anesthetic in the clinic because of its short induction time, small hemodynamic fluctuations, and rapid awakening. However, some studies have found that Sev is neurotoxic and can lead to cognitive dysfunction in older rats [29, 30]. Cognitive impairment caused by Sev anesthesia arises from intricate mechanisms, like neuroinflammatory activation, neurotransmitter homeostasis imbalance, mitochondrial dysfunction, and cytoplasmic calcium homeostasis imbalance, and Sev induces apoptosis in hippocampal neurons [31, 32]. In addition, the neurotoxicity of Sev was related to drug concentration and inhalation duration, with reference to pre-test results and published articles [26], in the present study, we used inhalation of 2% Sev for 5 h to construct a model of cognitive impairment in aged mice. After inhaling Sev for 5 h, the learning and spatial memory capabilities of the aged mice were significantly reduced, and the hippocampal tissues showed obvious damage leading to neuronal apoptosis, which indicated that the cognitive impairment model of aged mice was successfully constructed.

Mor is mainly found in Cornus officinalis and has anti-inflammatory, antioxidant, neuroprotective, osteoporosis preventive, and microvascular circulation improving effects [33–35]. Numerous studies have shown that neuroinflammation as a key mechanism associated with POCD [36, 37]. However, whether Mor ameliorates sev-induced neuroinflammation and cognitive deficits remains unknown. TNF-α is a classic inflammatory factor that promotes neuroinflammation by promoting microglial activation [38]. Additionally, in the immune response, pro-inflammatory factors such as IL-6 and IL-1β recruit immune cells to the infection or injury site to promote inflammation [39, 40]. Sun et al. [41] demonstrated that continuous inhalation of Sev caused heightened levels of inflammatory markers, such as TNF-α, IL-6, and IL-1β, in rat hippocampal tissue. In this study, inhalation of Sev resulted in elevated levels of IL-1β, TNF-α, and IL-6 in the hippocampal tissues, which is consistent with previous findings. The injection of Mor reduced the levels of inflammatory factors, suggesting that Mor inhibits Sev-mediated neuroinflammation. In addition, Mor significantly enhanced cognitive and spatial memory abilities in elderly mice, alleviated sev-induced hippocampal tissue damage and neuronal apoptosis, and ameliorated synaptic dysfunction, suggesting its potential for treating cognitive dysfunction.

The central nervous system houses microglia, which regulate neuroinflammation [42]. Microglia have two phenotypes, M1 and M2, and M1-type microglia, which release inflammatory cytokines and reactive oxygen species when activated, whereas M2-type microglia play a role in reducing inflammation and promoting tissue healing [43]. Sev has been reported to promote the activation of M1-type microglia and inhibit the conversion of microglia to M2-type, further exacerbating neuroinflammation [44]. Dai et al. [45] found that being exposed to 4.1% Sev for a duration of 6 h led to a significant increase in the levels of markers associated with M1-type microglia polarization, like CD86, iNOS, and interferon-γ, and downregulated the level of M2-type markers Transforming growth factor-β, CD206, and Arg-1. Similar results were found in our study, in which inhalation of Sev led to a notable increase in the proportion of M1-type microglia in the hippocampal tissues of aged mice. Importantly, the injection of Mor reversed the effect of Sev and promoted the transition of M1-type microglia to M2-type microglia, which may be an important mechanism by which Mor exerts anti-neuroinflammatory effects.

A growing body of research suggests that the activation of microglia and neuroinflammation may be linked to TLR4/NF-κB signaling and that blocking this signaling alleviates cognitive dysfunction [46, 47]. In addition, NF-κB signaling promotes the transcription of multiple inflammatory factors, with p65 serving as a pivotal component of this signaling cascade [48]. In the present study, inhalation of Sev elevated the expression of TLR4, consistent with the findings of Li et al. [49]. While Mor reduced the expression of TLR4, the molecular docking results confirmed that Mor and TLR4 could target binding in the form of hydrogen bonds. Injection of Mor reduced TLR4 protein and NF-κB p65 phosphorylation levels in mouse hippocampal tissues, further confirming that Mor inhibits the activation of the TLR4/NF-κB pathway. Additionally, the TLR4 agonist CRX-527 reversed the effects of Mor, suggesting that Mor ameliorates cognitive deficits in aged mice by blocking the TLR4/NF-κB pathway.

## Conclusion

In summary, our research demonstrates for the first time that Mor mitigates Sev-induced histopathological damage in the hippocampus of elderly mice, promotes the transformation of microglia into the M2 type, and suppresses neuroinflammation. Importantly, Mor blocked the TLR4/NF-κB pathway, suggesting that it may alleviate cognitive deficits in aged mice by modulating the TLR4/NF-κB pathway. This study elucidates the potential mechanism of action of Mor in alleviating cognitive impairment in aged mice, which provides a new reference for the clinical treatment of POCD. However, there are still some shortcomings of this research. Owing to time and condition constraints, this study had a small sample size. The effect of Mor on cognitive impairment in young mice and its mechanism of action should be explored further in the future. In addition, the signaling pathway regulatory network is complex, and the effects of Mor on other pathways associated with cognitive impairment need to be studied in depth.

## Supplemental data

**Highlights:**

1. Morroniside (Mor) ameliorates sevoflurane (Sev)-induced histopathological damage in the hippocampus of aged mice and alleviates synaptic dysfunction.

2. Mor inhibits apoptosis and neuroinflammation in mouse hippocampal neurons.

3. Mor promotes the conversion of mouse hippocampal microglia from the M1 to the M2 phenotype.

4. Mor binds to TLR4 targeting and inhibits TLR4/NF-κB pathway activation in hippocampal tissue.

5. TLR4 agonists attenuated the effect of Mor, suggesting that Mor ameliorates cognitive deficits in aged mice through hindering the TLR4/NF-κB pathway.

**Graphical abstract**


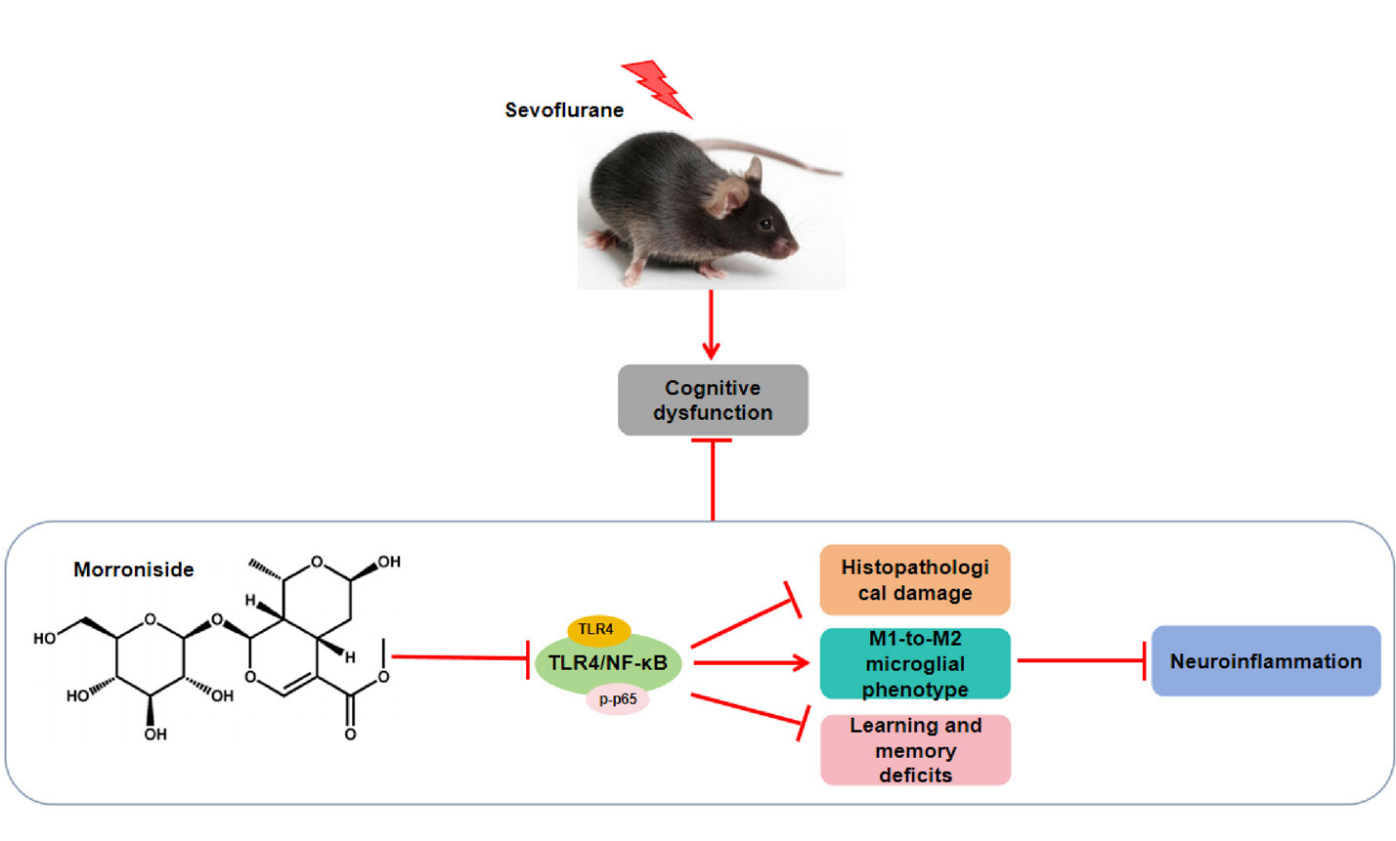


Mor blocked the TLR4/NF-κB pathway, alleviated hippocampal tissue damage and learning and memory deficits caused by Sev, induced the shift of microglia from M1 to M2 polarization, inhibited neuroinflammation, and thus improved cognitive impairment in aged mice.

## Data Availability

To acquire the data that underpin the results of this study, please contact the corresponding author.
